# Variables Associated With Negotiation Effectiveness: The Role of Mindfulness

**DOI:** 10.3389/fpsyg.2020.01214

**Published:** 2020-06-12

**Authors:** María C. Pérez-Yus, Ester Ayllón-Negrillo, Gabriela Delsignore, Rosa Magallón-Botaya, Alejandra Aguilar-Latorre, Bárbara Oliván Blázquez

**Affiliations:** ^1^Faculty of Education, Department of Psychology and Sociology, University of Zaragoza, Zaragoza, Spain; ^2^Institute for Health Research Aragón (IIS Aragón), Zaragoza, Spain; ^3^Faculty of Human Sciences and Education, Department of Psychology and Sociology, University of Zaragoza, Huesca, Spain; ^4^Faculty of Social and Labour Sciences, Department of Psychology and Sociology, University of Zaragoza, Zaragoza, Spain

**Keywords:** negotiation, mindfulness, emotional intelligence, personality, motivation, negotiation styles

## Abstract

Negotiation is the main mean of conflict resolution. Despite its capital importance, little is known about influencing variables or effective interventions. Mindfulness has shown to improve subjects’ performance in different settings but until now, no study has shown its impact in negotiation. The aim of this study is to analyze which variables are associated with effectiveness and to determine if meditators are more effective in negotiation. A cross-sectional descriptive study was carried out. The study variables were: socio-demographic variables, negotiation effectiveness (Negotiation Effectiveness Questionnaire), mindfulness (Five Facets of Mindfulness Questionnaire), emotional intelligence (Trait Meta-Mood Scale Questionnaire), personality (NEO-FFI personality inventory), motivation (McClelland Questionnaire), and negotiation style (Rahim Organizational Conflict Inventory-II). A correlational study and a multivariate model were developed. Negotiation effectiveness was associated with age, mindfulness, emotional intelligence, extraversion, openness, conscientiousness, achievement motivation, integrating, dominating, and compromising negotiation styles and inversely correlated toward neuroticism. The effectiveness of the negotiation is explained by the variables clarity, age, conscientiousness, dominating, and compromising style. Meditators were found to be more effective than non-meditators.

## Introduction

Conflict is part of our reality. It travels across different paths in our life, from the international and organizational settings to the most everyday spaces of the individual. And even though for some it implies pain, for others, its adequate resolution fosters their personal and professional development (Pimentel, 2013). One way to resolve conflicts is through negotiation. Munduate and Martínez (2003, p. 51), define negotiation as “a means of conflict resolution in which the parties wish to maintain or continue the exchange relationship, under new accepted foundations or conditions that are not yet determined.” Negotiation is conceived critical not only in the organizational setting but also in the personal lives of the individuals (Pruitt, 2013).

Given this setting, disentangling the factors that determine the effectiveness of negotiation is essential. Research on negotiation has a long trajectory in the study of the individual and social variables of negotiation effectiveness. Current views suggest the importance of an individual’s subjective construction in an interactive and evolving intrapersonal (e.g., mood and power), interpersonal (e.g., emotions), organizational (e.g., groups and culture), and virtual (e.g., computer-mediated negotiation) multilevel context (Bazerman et al., 2000; Thompson et al., 2010). Thus, psychological variables play a large role in negotiation effectiveness. However, the factors that account for negotiation effectiveness have yet to be clarified. As pointed out by Munduate and Medina (2013), a nuclear approach is to study the way that negotiators consider and handle conflict situations, identifying the aspects that should be taken into account when examining the effectiveness of negotiation meetings. According to Rodríguez et al. (2017, p. 246), “the negotiation process is effective when it resolves the conflict that gave rise to it, so that the parties perceive and express that the agreement is acceptable and satisfactorily includes the expectations of each of them”.

Negotiation styles have been related to negotiation effectiveness. Most authors divide bargainers into two groups: Cooperative/Problem-Solvers and Competitive/Adversarial. Cooperative bargainers tend to behave more pleasantly and strive to generate mutually beneficial agreements. Competitive bargainers are often less pleasant and they attempt to obtain optimal results for themselves. Most negotiators employ relatively “cooperative” or relatively “competitive” styles and they look forward to interactions with cooperative opponents. However, they tend to dread encounters with competitive adversaries (Craver, 2003). Schneider (2002) found that 60% of the adversarial bargainers were considered ineffective, while 75% of the problem-solving negotiators were considered effective. Adversarial bargainers who belittled, antagonized or conceived were more concerned with themselves than with their clients; meanwhile, problem-solving negotiators were able to balance assertiveness and empathy, “enlarging the pie” through creativity and flexibility, and understanding the other side through listening and perceptiveness. Negotiation effectiveness has been linked to the following negotiation styles (Rahim and Bonoma, 1979): Integrating (high interest in oneself and others), Obliging (low interest in oneself and high interest in others), Dominating (high interest in oneself and low interest in others), Avoiding (low interest in one’s own interests and those of others), and Compromising (intermediate between self-interest and that of others). Despite the apparent superiority of the cooperative style, the integrating style should be correspondingly superior to the other styles. Results, however, are contradictory (Bazerman and Neale, 1982; Butler, 1994; Cheung et al., 2006). Munduate et al. (1999), showed that the most effective negotiation style was a combination of compromising, integrating and dominating styles, and this pattern was more effective than any other style. Hence, it is now suggested that the overall effectiveness of each negotiation style will vary depending on the situation and time of the negotiation, so flexibility to adopt the most adaptive behavior seems crucial (Antonioni, 1998; Shell, 2001; Preuss and van der Wijst, 2017). Knowledge and management of negotiation style are essential for negotiators, and even experienced negotiators often appear to be unaware of their negotiation style (Miller, 2014).

Personality has been linked to workplace effectiveness and it is plausible that it may play a role in the effectiveness of negotiation (Sharma et al., 2018). For years, the so called “irrelevance consensus” advocated for the irrelevance of individual differences in the study of negotiation effectiveness (Sharma et al., 2013). Kyl-Heku and Buss (1996) showed that negotiators scoring high on surgency were more likely to put in extra time and effort, to make decisions for the group and not to conform to the beliefs of others; agreeable negotiators would do things for others without being asked and demonstrated care, courteousness, and politeness; disagreeable negotiators would exaggerate the faults of others and tamper with their work to make their own look better; conscientious negotiators would prioritize goals and effectively manage their time; finally, negotiators with high levels of intellect-openness would ask questions about many things, obtain a good education, and stand up for what they believed was right, despite opposition. Ma (2008) proposed a model in which negotiators scoring high on neuroticism and extraversion would be more likely to focus on face-saving; negotiators scoring high on agreeableness would be more likely to perceive negotiation as a win–lose (distributive) process and would demonstrate high levels of trust in the other negotiators; conscientiousness would not be related to win–lose orientation, face-saving or trust; and negotiators scoring high on openness to experience would be less likely to demonstrate a win–lose orientation. However, scientific literature on the relationship between personality and negotiation effectiveness reveals contradictory results (Antonioni, 1998; Barry and Friedman, 1998; Dimotakis et al., 2012; Monteiro et al., 2012).

Emotional intelligence (EI) has consistently been studied with regard to negotiation effectiveness. Emotions are fundamental in every relationship, including negotiation. Besides, managing emotions may not be straightforward for negotiators (Kelly and Kaminskienë, 2016). Furthermore, traditional IQ intelligence has received extensive attention, while emotions have been considered counterproductive in negotiation effectiveness (Kim et al., 2015). Fulmer and Barry (2004) created a model in which EI negotiators gather more and richer information about their opponent’s underlying interests, more accurately assess risk, use strategies that involve the manipulation of their own emotions or the emotions of their opponents, and induce desired emotions in these opponents. Empirical evidence does not reach consensus on the negotiation effectiveness of EI negotiators, who would have more positive experiences, or on the outcome satisfaction of their counterparts, trust, and desire to negotiate again. But these negotiators received lower objective scores than their counterparts, apparently benefiting in affective terms, but not in objective value (Der Foo et al., 2004; Mueller and Curhan, 2006; Kim et al., 2014). On the other hand, other authors have more consistently associated greater recognition, emotional understanding and emotional repair with negotiation effectiveness (Elfenbein et al., 2007; Pulido-Martos et al., 2013; Schlegel et al., 2018).

Motivation has also been examined with regard to negotiation effectiveness. Velden et al. (2007) showed that pro-self-motivation blocks decision making, thus harming the group. White (2008) found that higher motivation to participate encourages the use of negotiation strategies and resources to overcome constraints. Hubbard and Mannell (2001) found that better health and enjoyment motivations resulted in employees increasing their negotiation efforts. Epistemic motivation helped negotiators overcome a lack of information due to a larger information search, as opposed to heuristic trial and error (Van der Schalk et al., 2010; Ten Velden et al., 2010). Alexandris et al. (2017) found that both intrinsic and extrinsic motivation were associated with sport performance. As for negotiation styles, the need for power and egoistic motivation have been associated with competitive behaviors that maximize their own results without considering those of their opponent; whereas the need for affiliation and pro-social motivation have been linked to cooperation and the desire for fair distribution of both their own results and those of others (Schneer and Chanin, 1987; De Dreu et al., 2006). Although this heterogeneity of results suggests the potential role of motivation in negotiation effectiveness, this relationship remains obscure.

Furthermore, this study considers attention, the influence of mindfulness and, more specifically, the capacity of mindfulness on the effectiveness of the negotiation. In this sense, studies such as that of Brach (2008) show that the incorporation of facets of mindfulness such as purpose, presence, acceptance, and connectedness may improve the effectiveness of the negotiator and may make the experience more satisfactory and stimulating for the negotiator. Likewise, the practice of mindfulness may improve the negotiator and mediator’s capacity to listen closely to themselves and to others (Kuttner, 2008). On the other hand, Riskin (2002) points out that mindfulness practices can improve awareness and may dissociate negotiators from adverse thinking patterns filled with anxiety. Furthermore, Kopelman et al. (2012), show how acceptance without judgment and non-reaction to internal experience, permit the negotiator to reflectively benefit from adaptive cognitive and emotional resources and to behave strategically and effectively. On the other hand, the study by Reb and Narayanan (2014), examined how mindfulness influenced the results obtained in distributive negotiations. These authors reveal that the practice of conscious attention exercises prior to negotiations, could be an effective and economical way to improve the results of said negotiations. Furthermore, they suggest that this exercise could focus on the negotiation itself or could be a conscious attention practice that is unrelated to the situation. Being aware of the negotiation situation as well as his/her emotional reactions may allow the negotiator to better regulate their emotions (such as anxiety, greed, fear, and anger) and actions (such as the making of offers, counteroffers, and reactions to threats), and may allow the negotiators to carry out their intentions. The importance of analyzing this variable in relation to negotiation effectiveness is that it is a modifiable and trainable variable (Keng et al., 2012; Leventhal et al., 2015).

Inefficacy in negotiation can produce substantial and dramatic loses and most negotiators require practice and training in order to be effective (Movius, 2008). Hence, the literature has seen an increased number of studies considering the development of training programs to foster negotiation effectiveness (e.g., Bazerman and Neale, 1982; Taylor et al., 2008; Panke, 2012). It is necessary to conduct studies that further the knowledge of negotiation processes that could result in the development of interventions to improve negotiation effectiveness. In this study, in line with Sharma et al. (2013), we suggest that the study of individual variables is necessary in the field of negotiation effectiveness. We also hypothesize that people who meditate may be more effective negotiators and hence, meditation could be explored as a potential training technique to enhance negotiation effectiveness.

To our knowledge, no prior studies have addressed the collected variables within this study including the role of mindfulness in negotiation effectiveness. Therefore, the objectives of this study are two: on the one hand, to analyze which sociodemographic and psychological variables are associated with a higher effectiveness in negotiation and on the other hand, to determine if meditators are more effective in negotiating and the related variables.

The hypotheses of this study are:

1. Meditating people are more effective in negotiation and in associated variables such as EI.
2. There are socio-demographic variables that are associated with greater negotiation effectiveness.

## Materials and Methods

### Design

This is a descriptive, cross-sectional study, conducted between July of 2017 and July of 2018.

### Study Population and Sample Size

The population consisted of general population, including people who meditated. So, assuming an error of 5% with a confidence level of 95% and a precision of 5%, and adding 15% for potential mistakes in completing the questionnaires, at least 86 people were necessary. Finally, 94 subjects participated in the study, exceeding the necessary sample size. The sample consisted of 35 men and 59 women, with a mean age of 46.77 years (SD: 11.46), with 50 meditators and 44 non-meditators.

People meditator and non-meditator close to the researchers were invited to participate, including a broad spectrum in terms of age, gender, and education level. Participants were not selected. Subjects were invited to participate until the required sample size was reached, ensuring no statistical differences between meditators and non-meditators with regard to socio-demographic variables. All participants meeting the inclusion/exclusion criteria and answering the questionnaires were included in the study.

The inclusion criteria for the non-meditators were: Subjects over the age of 18; (a) who understand both spoken and written Spanish; (b) subjects give their informed consent; (c) who do not suffer from any type of disease affecting the Central Nervous System (TBI, dementia, cerebral organic pathology, etc.) or serious psychiatric diagnosis (substance dependence and abuse, schizophrenia or psychotic history, eating disorders, etc.); and (d) who do not consume any type of drug or medication that could affect the nervous system.

The inclusion criteria for the meditators were the same as the non-meditators, but they also had formal daily training for at least the past 6 months.

People from both groups who met the inclusion criteria were contacted, they were explained the research project, the ethical aspects of anonymity and confidentiality, and they were provided the questionnaire, either on paper or by email depending on the person’s preference. They were asked to fill it in at once and tell us how much time it had taken to complete it. Once they had completed it, they sent it to the research team, either on paper or by email. The response rate was 91%.

### Measurement

The principal study outcome was effectiveness in the negotiation, which was assessed through the Negotiation Effectiveness Questionnaire, based on the Mastenbroek (1987, 1991) model. This questionnaire was validated for the Spanish language by Serrano and Rodríguez (1993). A global Cronbach’s alpha of 0.807 was obtained, and 0.767, 0.551, 0.516, and 0.680 in the sub-scales of substantive results, power balance, constructive climate, and procedural flexibility, respectively. Except in subscales, balance of power and constructive climate, which indicate low consistency, the Cronbach’s alphas obtained indicate a good and moderate internal consistency. It consists of 40 items, assessed using a Likert scale between 1 and 5 (completely disagree to fully agree), evaluating the following four dimensions: substantive results (items 1 to 11), power balance (items 12 to 21), constructive climate (items 22 to 32), and procedural flexibility (items 33 to 40) (Munduate and Medina, 2013). A score for each individual dimension can be obtained and also a total score, that results from the sum of each subscale. The minimum and maximum scores for each dimension are: substantive results and constructive climate, between 11 and 55; balance of power, between 10 and 50; and procedural flexibility, between 8 and 40 points. The total score of the scale ranges between 40 and 200 points. A higher score implies greater effectiveness of the negotiation.

Other variables which, according to the literature, may be associated with the effectiveness of the negotiation, were also collected. These variables included: socio-demographic variables, EI, capacity for mindfulness, personality variables, motivation, and negotiation style.

The following socio-demographic variables were collected: gender (male/female), age, marital status (single, married or part of a couple, separated or divorced, widowed), whether or not they have children, education level (cannot read and write; able to read and write but no formal studies, primary school studies, secondary school studies, university studies, others), current employment status (student, housewife, unemployed with benefits, unemployed with no benefits, employee, employee with temporary work disability, permanent occupational disability, retired, others), and occupation coded according to the “British Registrar General” classification system (professionals, managers, higher level technicians, administrators and directors, technicians and associated professionals, group of sales and administration services personnel, skilled workers, unskilled workers, others).

Emotional intelligence was assessed using the Trait Meta-Mood Scale Questionnaire, consisting of 24 items (TMMS-24): The TMMS-24 is based on the Trait-Meta Mood Scale (TMMS) of the Salovey et al. (1995). The original scale is a feature scale that evaluates the metacognition of emotional states through 48 items. Specifically, the skills that we may be aware of within our own emotions as well as our ability to regulate them. The TMMS-24 consists of twenty-four items, with five options of different level of agreement with this item. The TMMS-24 contains three key dimensions of EI, with each of these dimensions containing eight (8) items. These dimensions are: 1. attention, which is defined as emotional perception, when the individual can feel and express his/her feelings appropriately. 2. Clarity, which is defined as the understanding of feelings and occurs when the individual knows their own emotional states well. 3. Repair, which would be equivalent to emotional regulation, when a person is able to properly regulate their emotional states. The first eight items correspond to the attention dimension, items 9 to 16 correspond to the dimension of clarity and the last 8 items correspond to the repair dimension. Each item ranges from a score of 1 (no agreement) to 5 (totally agree), therefore, each dimension has a range of between 8 and 40 points. The total score of the scale is obtained with the sum of all the items, there being no inverse items, therefore total score of the scale ranges between 24 and 120 points and each dimension has a range of between 8 and 40 points. The higher the score, the greater the EI. It was developed and validated in Spanish by Extremera et al. (2004), receiving a Cronbach’s alpha for the Attention and Clarity and Repair dimensions of 0.90 and 0.86, respectively. The Cronbach’s alphas obtained in this study have been 0.989 (total scale) and 0.847, 0.920, and 0.848 for the sub-scales of attention, clarity and repair, respectively. This indicates a good internal consistency.

The mindfulness variable was assessed using the shorter Spanish version of the Five Facets of Mindfulness Questionnaire (FFMQ-24). This version includes 24 items (Bohlmeijer et al., 2010), and was validated for the clinical population (with anxiety and depression symptomatology). However, for a general population of Europeans having good psychometric properties (α > 0.70) on all dimensions, a direct translation of the items from the original FFMQ validated in Spanish by Cebolla et al. (2012) was used. The items, which are answered on a Likert scale ranging from 1 (never or very rarely true) to 5 (very often or always true), referred to 5 facets or skills of mindfulness: (1) Observing; (2) Describing; (3) Acting with awareness; (4) Non-judging; and (5) Non-reacting to the internal experience. The five facets reveal a good internal consistency, both in the English version and in the Spanish validation used (Asensio-Martínez et al., 2019), with Cronbach’s alpha obtained ranged between 0.65 (“Observing” factor) and 0.80 (“Acting with awareness” factor). A global Cronbach’s alpha of 0.844 was obtained in this study, and 0.843, 0.853, 0.789, 0.834 and 0.795 were obtained in the sub-scales of observing, describing, acting with awareness, non-judging and non-reacting, which indicate a good internal consistency. There are direct and inverse scoring items. The Observing aspect has a minimum and maximum score that ranges from 4 to 20 points, while the other facets range from 5 to 25 points. The total score of the scale is obtained with the sum of all the facets, and ranges between 24 and 120 points. A higher score suggests a greater capacity for mindfulness in each of the facets.

The personality variable was assessed using the NEO-FFI personality inventory. This questionnaire, containing 60 items, is the short version of the NEO-PI-R (Costa and McCrae, 1989, 1992) and aims to evaluate the following five dimensions of personality: Neuroticism, Extraversion, Openness to experience, Agreeableness, and Conscientiousness. It is typically assumed that the NEO-FFI has the same psychometric properties as the complete instrument (Tokar et al., 1999; Murray et al., 2003). In fact, the few studies conducted on the psychometric qualities of this version show a high internal consistency and find that the five-factor structure is reproduced in all cases; however, some reagents have been found to have difficulties in their factor load and greater weakness in the Openness and Agreeableness factor. It was validated in Spanish by Aluja et al. (2005), obtaining a Cronbach’s alpha of 0.82, 0.78, 0.71, 0.71, 0.83 in the dimensions of Neuroticism, Extraversion, Openness, Agreeableness and Conscientiousness, respectively. In this study, a Cronbach’s alpha of 0.787 was obtained for the neuroticism factor, 0.821 in extraversion, 0.749 in openness, 0.728 in agreeableness and 0.762 for the conscientiousness factor, which indicate good and moderate consistency. The NEO-FFI is a comprehensive measure of personality traits that was constructed based on the general population, as used in this study, but it may also be used in clinical populations, and it is widely used on an international level. There are direct and inverse scoring items. The scores of each item are evaluated on a Likert scale between 1 and 5, so that each dimension can range between 12 (minimum score) and 60 (maximum score). A higher score suggests a greater trait in each of the evaluated dimensions.

Motivation was evaluated using the McClelland Questionnaire, which consists of 53 items distinguishing between the need for achievement, power or affiliation. Need for achievement is the urge to stand out, considering achievement based on a set of standards (norms), the struggle for success; the need for power would be the need to make others display certain behaviors that they would not display otherwise; the need for affiliation would be the desire for close, friendly and interpersonal relationships (Atkinson, 1964; McClelland, 1985). It was developed and validated by Sudarsky and Cleves (1976), who analyzed reliability using the Kunder–Richardson index (KR20), obtaining 0.85 in achievement motivation, 0.78 in power motivation and 0.85 in affiliation motivation. Each item has three response options, each of which is oriented toward each type of motivation. A score is obtained in each type of motivation, based on the number of responses oriented to each motivation. Therefore, the minimum and maximum score that can be obtained for each type of motivation ranges from 0 to 53. The higher the score, the greater the orientation toward that type of motivation.

Negotiation style was evaluated using the Rahim Organizational Conflict Inventory-II (ROCI-II) (Rahim, 1983a,b), which has three types (A, B, and C), referred to subordinates, supervisors and partners. For this study, version C (relationship with colleagues) was chosen. This questionnaire, consisting of 28 items, is based on the conceptualization of the conflict leadership style elaborated by Rahim and Bonoma (1979), which distinguishes between two dimensions: self-interest and the interest of others, obtaining five possible styles: integrating, avoiding, obliging, dominating, and compromising. The Spanish validation was created by Munduate et al. (1993), obtaining a Cronbach’s alpha of 0.77 in the integrating dimension, 0.76 in servility, 0.75 in domination, 0.70 in avoiding and 0.62 in compromising. In this study, a Cronbach’s alpha of 0.877 was obtained for the integrating style, 0.559 in avoiding, 0.759 in obliging, 0.721 in dominating, and 0.742 in compromising. Except in the avoiding subscale, the Cronbach’s alphas obtained indicate a good-moderate internal consistency. Each item is evaluated on a Likert scale ranging from 1 to 5, with a higher score suggesting a greater tendency to use a certain style during negotiation. In the Spanish validation, the minimum and maximum scores in each style are as follows: in integrating style, between 7 and 35 points, avoiding between 6 and 30 points, dominating and obliging, between 5 and 25 points, and finally, compromising between 3 and 15 points.

The following data were also collected from the meditators: practice time (months), time spent meditating during 1 week, and time allocated during the week to each type of practice (3 min practice, breathing, body scan, walking, movement, values and goodness).

### Statistical Analysis

All of the collected variables followed a normal distribution according to the Shapiro–Wilk statistic, except for the following: month of practice in the meditators, observation, integration, servility, commitment, and flexibility. Therefore, non-parametric statistics were used to analyze these variables.

First, in response to the objective of determining whether the meditators are more effective in the negotiation, a description of the sample was made for the study variables, using percentages of the qualitative variables, and means, standard deviations in the variables quantitative. Cronbach’s alpha was calculated for each questionnaire and dimension to determine the reliability of the collected data. A comparison of the groups was also performed to analyze if they were comparable with respect to the socio-demographic variables, and with respect to the variable of effectiveness of the negotiation, and variables that may be associated with the effectiveness of negotiation: EI, capacity for mindfulness, personality, motivation, and negotiation styles. To do this, we used the Student’s T statistic for the variables following a normal distribution and the non-parametric statistic (Mann–Whitney U test) for those variables that did not have a normal distribution.

In response to the objective of determining which variables are associated with the effectiveness of the negotiation, an analysis of correlations between the effectiveness variable of the negotiation and the quantitative variables was conducted, using Pearson’s P and Spearman’s Rho, depending on whether the variable had a normal distribution or not. The effectiveness of the negotiation was also analyzed, according to the different categories of the qualitative variables, using the Chi-square statistic. Finally, a multivariate model was developed to identify the predictive variables of greater negotiation effectiveness. The independent variables having a significant correlation were introduced in the regression model following a stepwise method (Hamilton, 1994) and a final model was obtained. Prior to multiple regression, a correlation analysis was carried out between the variables that were to be introduced in the model. An association between EI and mindfulness was obtained (clarity and describing *p*-value < 0.001, repair and describing *p*-value 0.039). But since they did not obtain significance in the comparison of EI between meditators and non-meditators, they were introduced into the model. However, the regression was also performed, without including the mindfulness variables and the results in terms of the significant variables were the same. The total score for EI and mindfulness was not included in the model, since these are obviously related to the construct factors that they measure.

The significance value was considered when the *p*-value was less than 0.005. The SPSS 21 program was used to conduct the statistical analyses.

### Ethical Issues

The study was evaluated by the Ethical Research Committee of Aragon, Spain, and it was determined that evaluation was not necessary (15/2017). However, the study was conducted in accordance with the Helsinki Declaration. All of the subjects filled out an informed consent form, and their data was anonymized.

## Results

### Characteristics of the Sample

Ninety-four subjects participated in the study, consisting of 59 women and 35 men. Table 1 shows the description of the total sample according to the socio-demographic study variables. The participant profile is a woman whose mean age is 47, married, with university studies, and employed in a professional, senior technician, or managerial position.

**TABLE 1:** Description of the total sample according to the socio-demographic study variables and the following variables: emotional intelligence, mindfulness, personality, motivation, negotiation styles, and effectiveness of the negotiation.

**Variables**	**Percentage**
	**mean (SD)**
**Gender**	
Male	37.2%
Female	62.8%
Age	46.77 (11.46)
**Marital status**	
Single	24.5%
Married or in couple	59.6%
Separated or divorced	14.9%
Widowed	1.1%
**Children**	
Yes	50.5%
No	49.5%
**Education level**	
Primary studies	2.2%
Secondary studies	7.4%
University studies	90.4%
Others	0%
**Employment status**	
Student	4.3%
Housewife	0%
Unemployed with benefits	0%
Unemployed without benefits	1.1%
Employee	87%
Employee with TWD	0%
Permanent disability	6.5%
Retired	1.1%
Others	0%
**Occupation**	
Professionals, managers, higher level technicians	60%
Administrators and directors	7.7%
Technicians and associated professionals	13.8%
Sales and administration services personnel	18.5%
Skilled workers	0%
Unskilled workers, others	0%
**Emotional intelligence**	
Attention	18.70 (4.20)
Clarity	20.34 (4.36)
Repair	21.27 (4.09)
Total score	60.31 (9.63)
**Mindfulness**	
Observing	15.07 (3.39)
Describing	18.97 (3.63)
Acting with awareness	18.31 (3.31)
Non-judging	17.77 (3.65)
Non-reacting	18.59 (4.16)
Total score	88.70 (11.39)
**Personality**	
Neuroticism	28.74 (7.88)
Extraversion	41.65 (6.72)
Openness to experience	43.02 (6.60)
Agreeableness	45.27 (6.05)
Conscientiousness	43.53 (6.14)
**Motivation**	
Achievement	25.48 (4.77)
Power	6.03 (2.35)
Affiliation	21.01 (4.77)
**Negotiatión styles**	
Integrating	29.66 (3.65)
Avoiding	17.76 (3.51)
Obliging	19.02 (3.79)
Dominating	13.00 (3.25)
Compromising	15.71 (1.81)
**Effectiveness of negotiation**	
Substantive results	31.37 (5.38)
Power balance	30.53 (3.69)
Constructive climate	43.94 (4.27)
Procedural flexibility	29.87 (3.22)
Total score	135.70 (10.09)

*TWD, temporary work disability.*

### Variables Associated With the Effectiveness of the Negotiation

In order to analyze the variables that are associated with the effectiveness of the negotiation, we first analyzed the effectiveness of the negotiation in relation to the different categories of the qualitative socio-demographic variables, without obtaining statistically significant differences (Kendall’s Tau B for effectiveness-gender = 0.858, effectiveness-civil status = 0.218, effectiveness-children = 0.263, effectiveness-level of studies = 0.680, effectiveness-work situation = 0.862, effectiveness-category employment = 0.984). As for the analysis of the relationship of the quantitative variables and the effectiveness of the negotiation, its correlation was calculated. These results are shown in Table 2. As it shows, there is a direct correlation with age, all of the EI variables, the capacity for mindfulness (observing, describing, and non-reacting), the personality of extraversion, openness and conscientiousness, achievement motivation, the style of integrating negotiation, dominating and compromising. On the other hand, an inverse correlation exists with the personality variable of neuroticism. No correlation exists with the months of meditative practice.

**TABLE 2:** Correlation between effectiveness of the negotiation and the following variables: emotional intelligence, mindfulness, personality, motivation, and negotiation styles.

**Variables**	**P Pearson/Spearman**
Age	0.301**
**Emotional intelligence**	
Attention	0.264*
Clarity	0.476**
Repair	0.336**
Total score	0.472**
**Mindfulness**	
Observing	0.286**
Describing	0.343**
Acting with awareness	0.174
Non-judging	–0.112
Non-reacting	0.194
Total score	0.278**
**Personality**	
Neuroticism	−0.248*
Extraversion	0.225*
Openness to experience	0.332**
Agreeableness	0.099
Conscientiousness	0.400**
**Motivation**	
Achievement	0.223*
Power	0.077
Affiliation	–0.203
**Negotiatión styles**	
Integrating	0.337**
Avoiding	–0.052
Obliging	0.147
Dominating	0.254*
Compromising	0.380**
Months of practice	0.201

* *p*-value < 0.05, ** *p*-value < 0.01. Spearman’s statistic was used for observation, integration, servility, and tendency to compromise.

Regarding the linear regression model, five models were obtained, but Table 3 shows the model with a greater explanatory capacity for greater effectiveness of the negotiation. In this model, the effectiveness of the negotiation is explained by the variables of clarity, age, conscientiousness, dominating, and compromising, with this last variable obtaining a significant weight. This model explains 54.5% of the model’s variance.

**TABLE 3:** Linear regression on the variables influencing the effectiveness in the negotiation.

**Effectiveness of the negotiation**	**Coefficient**	**p-value**	**Confidence interval 95%**	
			**Lower**	**Higher**
Constant	62.102	<0.001	44.392	79.812
Clarity	0.692	<0.001	0.298	1.085
Age	0.207	0.004	0.070	0.344
Conscientiousness	0.451	0.002	0.174	0.728
Dominating	0.669	0.006	0.208	1.190
Compromising	1.326	0.004	0.426	2.225
R^2^	0.495			
R^2^ adj	0.465			

### Role of Mindfulness in the Effectiveness in the Negotiation

Of the 94 participants in the study, 50 subjects were meditators (53.2%) and 44 were non-meditators (46.8%). The meditators spent an average of 8.9 years meditating (107.17 months, SD 108.68, median: 60 months); they engaged in a mean of 263.26 min per week (SD: 240.14), devoting more time to practices of movement, breathing, and values.

Table 4 shows the comparison between meditators and non-meditators with respect to the socio-demographic variables and the variables of EI, mindfulness, personality, motivation, negotiation styles, and negotiation effectiveness. As seen, no differences exist between the two groups in the socio-demographic variables, so it may be considered that they are comparable in these parameters, but they do reveal significant differences in the variables of effectiveness of the negotiation and related variables. Specifically, meditators reveal significant differences in the following variables: greater clarity (EI); greater mindfulness (in total score and the facets of observing, describing, acting with awareness, non-judging, and non-reacting); less neuroticism but greater openness to experience, agreeableness, and conscientiousness; a greater tendency to acquire an integrating style in the negotiation; and a greater effectiveness of the negotiation (in the total score and in the factors of power balance and constructive climate).

**TABLE 4:** Comparison of the sample of meditators and non-meditators based on the socio-demographic study variables and the variables emotional intelligence, mindfulness, personality, motivation, negotiation styles, and negotiation effectiveness.

**Socio-demographic variables**	**Percentage/mean (SD)**		
	**Meditators**	**Non meditators**	***p*-value**
	***N* = 50**	***N* = 44**	
**Gender**			
Male	36%	38.6%	0.792
Female	64%	61.4%	
Age	48.66 (10.00)	44.61 (12.70)	0.093
**Marital Status**			
Single	24%	25%	0.136
Married or in couple	52%	68.2%	
Separated or divorced	22%	6.8%	
Widowed	2%	0%	
**Children**			
Yes	56%	44.2%	0.256
No	44%	55.8%	
Education level			
Primary studies	0%	4.5%	0.309
Secondary studies	8%	6.8%	
University studies	92%	88.7%	
Others	0%	0%	
**Employment status**			
Student	4.2%	4.5%	0.730
Housewife	0%	0%	
Unemployed with benefits	0%	0%	
Unemployed without benefits	0%	2.3%	
Employee	87.5%	86.4%	
Employee with TWD	0%	0%	
Permanent disability	6.3%	6.8%	
Retired	2.1%	0%	
**Occupation**			
Professionals, managers, higher level technicians	69.4%	48.3%	0.126
Administrators and directors	8.3%	6.9%	
Technicians and associated professionals	13.9%	13.8%	
Sales and administration services personnel	8.3%	31%	
Skilled workers	0%	0%	
Unskilled workers, others	0%	0%	
Variables related to negotiation			
Emotional intelligence			
Attention	18.42 (4.43)	19.02 (3.95)	0.491
Clarity	21.48 (4.38)	19.05 (4.01)	0.006
Repair	21.78 (4.00)	20.68 (4.16)	0.197
Total score	61.68 (9.35)	58.75 (9.81)	0.143
**Mindfulness**			
Observing	16.10 (2.46)	13.91 (3.92)	0.009
Describing	20.10 (3.13)	17.68 (3.76)	0.001
Acting with awareness	19.16 (3.26)	17.34 (3.13)	0.007
Non-judging	18.18 (3.50)	17.30 (3.79)	0.246
Non-reacting	20.14 (3.68)	16.82 (4.01)	<0.001
Total score	93.88 (10.14)	83.05 (10.11)	<0.001
**Personality**			
Neuroticism	26.36 (7.43)	31.45 (7.58)	0.001
Extraversion	42.00 (6.45)	41.25 (7.07)	0.595
Openness to experience	45.30 (5.19)	40.43 (6.84)	<0.001
Agreeableness	47.52 (5.10)	42.70 (6.07)	<0.001
Conscientiousness	45.00 (5.47)	41.86 (6.50)	0.014
**Motivation**			
Achievement	20.49 (4.31)	21.60 (5.23)	0.272
Power	5.96 (2.39)	6.11 (2.33)	0.754
Affiliation	26.37 (5.36)	24.50 (5.82)	0.113
Negotiatión styles			
Integrating	30.38 (3.67)	28.84 (3.48)	0.023
Avoiding	17.54 (2.91)	18.00 (4.21)	0.545
Obliging	19.60 (2.79)	18.36 (3.87)	0.070
Dominating	12.42 (2.95)	13.66 (3.48)	0.060
Compromising	15.98 (1.66)	15.41 (1.94)	0.173
**Efficacy in negotiation**			
Substantive results	31.54 (5.77)	31.16 (4.96)	0.736
Power balance	31.84 (3.04)	29.00 (3.82)	<0.001
Constructive climate	44.88 (4.09)	42.84 (4.24)	0.0021
Procedural flexibility	30.32 (3.24)	29.35 (3.16)	0.142
Total score	138.58 (8.90)	132.35 (10.45)	0.003

*TWD, temporary work disability. Statistic used: Student’s T, except in the variables of observation, integration, servility, tendency to compromise, and flexibility, for which the Mann–Whitney U was used.*

## Discussion

This study is the first to analyze the relationship between the practice of meditation and the effectiveness of negotiation. Meditators are found to have a greater negotiating effectiveness than their non-meditating counterparts having similar socio-demographic characteristics. This has been found to be significant for the dimensions of power balance and constructive climate, although in all cases meditation has been shown to favor the effectiveness of negotiation. No significant differences have been found for substantive results and procedural flexibility.

As mentioned in the introduction, negotiation emerges as an effective method of resolving conflicts. So, in this situation of conflict we find a great variety of emotions which should be considered individually: disgust, pleasure, surprise, fear, and anger. On the other hand, during negotiation, the emotions that each person expresses may or may not be perceived by their counterpart. And furthermore, negotiation is a setting in which success depends on the ability to communicate, exchange information and make accurate social judgments. As revealed by Elfenbein et al. (2007), effective negotiation requires that each of the parties develop an understanding of the interests and preferences of their counterparts, as well as the precise recognition of one’s emotions. For these reasons, the ability to deal with subtle communication signals may be beneficial to negotiators, and may help guide or prevent possible blockage.

Thus, the greater negotiating effectiveness of the meditators may be related to their greater capacity for mindfulness as demonstrated in this study in all the dimensions except for non-judgement. These dimensions, in turn, correlated positively with negotiating effectiveness. The greater capacity of observation and the awareness of the physical stimuli, thoughts, feelings and bodily sensations involved in a negotiation process, including one’s own interests and those of others, can lead them to having an attitude of “firm flexibility” when negotiating, by acting with awareness and without reacting with “automatic pilot” processes (Baer et al., 2006), characterized by intermediate positions between pleasing one’s own and others’ interests in obtaining substantive results, between obliging and dominating in relation to the power of each party, fostering a constructive climate of joviality and greater openness and flexibility in the search for solutions, as shown by the superior results obtained by the meditators in the dimensions of effectiveness in negotiation.

On the causes of this greater negotiating effectiveness of the meditators versus the non-meditators, the linear regression model obtained in this study has included the variables compromising, clarity, dominating, conscientiousness, and age. Contrary to our hypotheses, the variable of mindfulness has not shown explanatory power in this study. This leads us to reflect on the existence of other variables that mediate the relationship between meditation and the greater effectiveness of negotiation as demonstrated by this group. With the exception of age and dominating, in which there were no significant differences with regards to meditating, the remaining variables have been higher in the group of meditators, who, have been significantly related to greater negotiating effectiveness. Age is a frequent variable in studies conducted on negotiating effectiveness. This variable may generally affect the effectiveness of all of the subjects of this study, since both meditator and non-meditator groups did not differ in it. It is expected that older age correlates with greater experience and negotiating effectiveness.

The variable having the greatest explanatory power in our study on negotiating effectiveness was the compromising negotiation style. This negotiation style implies an intermediate position of the subjects between satisfaction with their own interests and those of others, yielding through the exchange of concessions or the search for intermediate solutions in order to reach a solution that is acceptable by all parties (Rahim and Bonoma, 1979). Mindfulness has been defined as one of the three components of compassion (Neff, 2003). Compassion consists of kindness in terms of understanding one’s own suffering and that of others in the face of criticism, common humanity or the knowledge that suffering is shared and that it counteracts isolation, and mindfulness, the awareness of suffering and not over-identifying with it. Accordingly, meditators have greater compassion toward themselves and toward others, which in a negotiation situation implies greater interest in themselves and in others. Our results support this hypothesis since meditators were better at compromising, obliging and significantly at integrating all of the styles in which the interest in the other party is greater, and on the contrary, they were showed to be less likely to present the avoiding and dominating styles, in which this interest is low.

In line with the results of our study and others (Munduate et al., 1993; Cheung et al., 2006; Luque et al., 2014), the integrating and compromising negotiation styles significantly correlated with negotiating effectiveness. Likewise the dominating style, in our study correlated significantly with negotiating effectiveness, but not obliging and avoiding. In the study by Cheung et al. (2006), dominating also presented this relationship (Cheung et al., 2006), while avoiding and obliging had less influence on achieving functional negotiation results. The diverse effectiveness of certain apparently conflicting styles has been described as context-dependent, so styles where the interest in the other party predominates may improve long-term negotiations, while domination could be more effective in more immediate contexts. In addition, results show that patterns using multiple conflict handling styles were more effective than patterns based on one single style (Munduate et al., 1999).

Second in terms of importance in the regression model of this study, is the clarity subscale of EI, which is defined as the understanding of feelings and occurs when the individual knows their own emotional states well. Meditators were significantly superior in this attribute according to the extensive bibliography that supports the relationship between mindfulness and EI (Thomas, 2006; Schutte and Malouff, 2011). In general, all facets of EI correlated significantly with negotiating effectiveness. These results coincide with those obtained in the study by Mueller and Curhan (2006), which analyzes each dimension of EI separately and explores how each of these dimensions can contribute to the successful outcome of the opposing party in a negotiation. And it reveals that a convincing correlation exists between the ability to understand the emotion and the mood of the opposing party in a negotiation. Furthermore, in contexts involving future negotiations, the ability to induce positive moods in others is a skill that negotiators with a clear understanding of emotions should consider.

To continue with our model, one facet of the personality model handled in this study (Murray et al., 2003), conscientiousness, is found to be important. Known as “being conscious,” is characterized by self-control of impulses and planning, organization and execution of tasks, involves careful planning and persistence in goals and is also called “achievement will.” Meditators were significantly superior in this construct, and in the dimensions of openness and agreeableness, with no differences being found in extraversion, and significantly less in neuroticism. In this sense, agreeableness reflects the interpersonal tendency of an individual to be characterized as altruistic, considerate, kind, generous, trusting, helpful, and supportive. As expected, meditators scored significantly higher in this construct than non-meditating subjects.

Coinciding with other studies (Yiu and Lee, 2011; Monteiro et al., 2012), extraversion, openness to experience and conscientiousness have shown significant relationships as influential personality variables in the effectiveness of negotiation. As the study by Sharma et al. (2013) shows, extroverts have a sociable nature, and therefore they may reveal more information about their own preferences and alternatives to the agreement during negotiations. Individuals having a greater openness tend to display greater flexibility and divergent thinking and this could help broaden negotiations to develop better offers for themselves and for others. And, negotiators who are more aware tend to outperform their less aware peers, since they tend to achieve more tasks and make more preparations in complex tasks. As has been widely observed in other studies, neuroticism has a negative and significant relationship with negotiation effectiveness. As the study by Barry et al. (2004) shows, neuroticism is related to negative emotions such as hostility and depression. Furthermore, this dimension has been associated with a greater susceptibility of the negative mood and with a greater memory of negative words and negative judgments. Therefore, in a negotiation situation, this will influence the choice of tactics and the strategy chosen and, of course, how the meeting will evolve, the overall satisfaction of the negotiation process and the subsequent agreement fulfillment. Agreeableness has not revealed any significant relationships. According to Sharma et al. (2013), this may be because the most pleasant people tend to value relationships more and to avoid conflict (Graziano et al., 1996). And they have more motivation to achieve interpersonal intimacy, leading to less assertive tactics in a negotiation environment (Cable and Judge, 2003).

### Practical Implications of the Use of Mindfulness in Negotiation

In this study, the practice of meditation has been found to be beneficial to a greater effectiveness of negotiation. Currently, mindfulness programs for negotiation already exist, although until now, no study has proven its effectiveness (Riskin, 2004; Awaida, 2013).

When a person practices meditation, it may not only improve their personal life but also their professional life. Various studies have suggested that this practice leads to improvements in negotiation skills (Brach, 2008; Kuttner, 2008), because when mindfulness is practiced, mindfulness increases, and awareness is achieved, not only of the verbal language of the counterpart, but also of their non-verbal communication, so vital in negotiation. Thus, the importance of the influence of body language, physical contact, eye contact, facial expression, gestures, and body posture, in the negotiation processes (Gordillo et al., 2014) has been studied. In addition, mindfulness allows a negotiator to sustain attention over time (Chiesa et al., 2011). Conscientious negotiators may be better able to keep their mind on the task at hand, avoiding distractions. This may allow them to process more verbal and non-verbal signals from the counterpart, as compared to less aware negotiators (Reb and Narayanan, 2014).

Furthermore, along with this externally focused attention, an increase in internally focused attention may permit negotiators a greater awareness of their emotional and visceral reactions that arise during negotiation. This, in turn, may allow them to make better decisions from an ethical point of view (Ruedy and Schweitzer, 2010). It may also allow them to regulate their emotions better, based on the specific needs of the negotiation situation (Reb and Narayanan, 2014). According to Kaplan (2016), the individual who meditates becomes aware of what is truly important, and learns to be honest to get what they really want in a negotiation situation. Over time, this leads to feeling less attached to the results. And, as a result, the people with whom you interact and negotiate feel less coerced and freer to make a decision that may be mutually beneficial.

Some studies have shown a positive relationship between mindfulness and empathy (Dekeyser et al., 2008; Greason and Cashwell, 2011). Schure et al. (2008) show that the practice of meditation increases empathy. Therefore, if meditation makes you feel more empathetic, it will also transform the way you relate to people in a negotiation situation. When the negotiator is empathetic, they understand the perspective of the other party so they send the message that they are committed to the negotiation and the relationship, an integral step in the creation of trust. This fluency also indicates that they are willing to move forward with their negotiated agreement (Malhotra, 2004).

This article has both strengths and limitations. Of its strengths is that it considers a topic, like the relationship between mindfulness and negotiation, which until now has not been widely studied, considering the fact that negotiation is a very common process in our daily lives. Among the limitations, there are the design of the study, since it is a cross-sectional study, causality can not be inferred, and also the limited sample size of this study. Another limitation of the study is that, when performing the bivariate analysis between the variables that were to be introduced in the model, a relationship between mindfulness and EI was found (clarity and describing, repair and describing). However, the results of the multivariate analysis were the same when including or not including the variables of mindfulness (observing, describing), suggesting the stability of the obtained model. Further studies with larger samples sizes that permit greater statistical analysis may help to shed additional light on this subject of study.

## Conclusion

In conclusion, it may be considered that the negotiator who meditates may manage to create confidence, focus on the actions and the correct words, not fixate on the results and maintain an order in his/her mind. All of these skills are associated with an effective negotiation.

## Data Availability Statement

The datasets generated for this study are available on request to the corresponding author.

## Ethics Statement

The studies involving human participants were reviewed and approved by the Ethical Research Committee of Aragon, Spain (15/2017). The patients/participants provided their written informed consent to participate in this study.

## Author Contributions

BO, MP-Y, and EA-N contributed conception and design of the study. BO organized the database and performed the statistical analysis. MP-Y and EA-N wrote the first draft of the manuscript. RM-B, AA-L, GD, BO, EA-N, and MP-Y wrote sections of the manuscript. All authors contributed to manuscript revision and read and approved the submitted version.

## Conflict of Interest

The authors declare that the research was conducted in the absence of any commercial or financial relationships that could be construed as a potential conflict of interest.

## References

[B1] Alexandris K. Du J. Funk D. Theodorakis N. D. (2017). Leisure constraints and the psychological continuum model: a study among recreational mountain skiers. *Leisure Stud.* 36 670–683. 10.1080/02614367.2016.1263871

[B2] Aluja A. García O. Rossier J. García L. (2005). Comparison of the neo-ffi, the neo-ffi-r and an alternative short version of the neo-pi-r (neo-60) in Swiss and Spanish samples. *Personali. Indvid. Differ.* 38 591–604. 10.1016/j.paid.2004.05.014

[B3] Antonioni D. (1998). Relationship between the big five personality factors and conflict management styles. *Int. J. Confl. Manag.* 9 336–355. 10.1108/eb022814

[B4] Asensio-Martínez Á Masluk B. Montero-Marin J. Olivan-Blázquez B. Navarro-Gil M. T. García-Campayo J. (2019). Validation of five facets mindfulness questionnaire – short form, in Spanish, general Health Care Services patients sample: prediction of depression through mindfulness scale. *PLos One* 14:e0214503. 10.1371/journal.pone.0214503 30939151 PMC6445454

[B5] Atkinson J. (1964). *Introduction to Motivation.* New York, NY: Van Nostrand.

[B6] Awaida J. (2013). “Executive master in consulting and coaching for change,” in *Negotiating Mindfully. A Constructivist Grounded Theory Approach Proposing a Conceptual Framework Linking Mindfulness to Negotiation Effectiveness*, (Fontainebleau: INSEAD).

[B7] Baer R. A. Smith G. T. Hopkins J. Krietemeyer J. Toney L. (2006). Using self-report assessment methods to explore facets of mindfulness. *Assessment.* 13 27–45. 10.1177/1073191105283504 16443717

[B8] Barry B. Friedman R. A. (1998). Bargainer characteristics in distributive and integrative negotiation. *J. Personal.Soc. Psychol.* 74 345–359. 10.1037/0022-3514.74.2.345

[B9] Barry B. Fulmer I. S. Van Kleef G. A. (2004). “I laughed, I cried, I settled: the role of emotion in negotiation,” in *The handbook of negotiation and culture*, eds Gelfand M. J. Brett J. M. (Redwood City, CA: Stanford Business Books).

[B10] Bazerman M. H. Curhan J. R. Moore D. A. Valley K. L. (2000). Negotiation. *Annu. Rev. Psychol.* 51 279–314. 10.1146/annurev.psych.51.1.279 10751973

[B11] Bazerman M. H. Neale M. A. (1982). Improving negotiation effectiveness under final offer arbitration: the role of selection and training. *J. Appl. Psychol.* 67 543–548. 10.1037/0021-9010.67.5.543

[B12] Bohlmeijer E. Prenger R. Taal E. Cuijpers P. (2010). The effects of mindfulness-based stress reduction therapy on mental health of adults with a chronic medical disease: a meta-analysis. *J. Psychos. Res.* 68 539–544. 10.1016/j.jpsychores.2009.10.005 20488270

[B13] Brach D. (2008). A logic for the magic of mindful negotiation. *Negot. J.* 24 25–44. 10.1111/j.1571-9979.2007.00165.x

[B14] Butler J. K. (1994). Conflict styles and outcomes in a negotiation with fully-integrative potential. *Int. J. Confl. Manag.* 5 309–325. 10.1108/eb022749

[B15] Cable D. M. Judge T. A. (2003). Managers’ upward influence tactic strategies: the role of manager personality and supervisor leadership style. *J. Organ. Behav.* 24 197–214. 10.1002/job.183

[B16] Cebolla A. García-Palacios A. Soler J. Guillen V. Baños R. Botella C. (2012). Psychometric properties of the Spanish validation of the Five Facets of Mindfulness Questionnaire (FFMQ). *Eur. J. Psychiatry* 26 118–126. 10.4321/S0213-61632012000200005 31832788

[B17] Cheung S. O. Yiu T. W. Yeung S. F. (2006). A study of styles and outcomes in construction dispute negotiation. *J. Constr. Eng. Manag.* 132 805–814. 10.1061/(ASCE)0733-93642006132:8(805)

[B18] Chiesa A. Calati R. Serretti A. (2011). Does mindfulness training improve cognitive abilities? A systematic review of neuropsychological findings. *Clin. Psychol. Rev.* 31 449–464. 10.1016/j.cpr.2010.11.003 21183265

[B19] Costa P. McCrae R. (1989). *The Neo-pi / Neo-ffi Manual Supplement.* Odessa, FL: Psychological Assessment Resources.

[B20] Costa P. McCrae R. (1992). *Revised Neo Personality Inventory (neo pi-r) and neo Five-Factor Inventory (neo-ffi) Professional Manual.* Odessa, FL: Psychological Assessment Resources.

[B21] Craver C. B. (2003). Negotiation styles: the impact on bargaining transactions. *J. Dispute Resol.* 48:328.

[B22] De Dreu K. W. Beersma B. Stroeme K. Euwena M. C. (2006). Motivated information processing, strategic choice, and the quality of negotiated agreement. *J. Personal. Soc. Psycholo.* 90 927–943. 10.1037/0022-3514.90.6.927 16784343

[B23] Dekeyser M. Raes F. Leijssen M. Leysen S. Dewulf D. (2008). Mindfulness skills and interpersonal behaviour. *Personal. Indvid. Differ.* 44 1235–1245. 10.1016/j.paid.2007.11.018

[B24] Der Foo M. Elfenbein H. A. Tan H. H. Aik V. C. (2004). Emotional intelligence and negotiation: the tension between creating and claiming value. *Int. J. Confl. Manag.* 15 411–429. 10.1108/eb022920

[B25] Dimotakis N. Conlon D. E. Ilies R. (2012). The mind and heart (literally) of the negotiator: personality and contextual determinants of experiential reactions and economic outcomes in negotiation. *J. Appl., Psychol.* 97 183–193. 10.1037/a0025706 21967294

[B26] Elfenbein H. A. Foo M. D. White J. Tan H. H. Aik V. C. (2007). Reading your counterpart: the benefit of emotion recognition accuracy for effectiveness in negotiation. *J. Nonverb. Behav.* 31 205–223. 10.1007/s10919-007-0033-7

[B27] Extremera N. Fernández-Berrocal P. Mestre J. M. Guil R. (2004). Medidas de evaluación de la inteligencia emocional. *Rev. Latinoam. Psicol.* 36:2.

[B28] Fulmer I. S. Barry B. (2004). The smart negotiator: cognitive ability and emotional intelligence in negotiation. *Int. J. Confl. Manag.* 15 254–272.

[B29] Gordillo F. López R. Mestas L. Corbi B. (2014). Comunicación no verbal en la negociación: la importancia de saber expresar lo que se dice. *Rev. Electron. Psicol.* 17 646–666.

[B30] Graziano W. G. Jensen-Campbell L. A. Hair E. C. (1996). Perceiving interpersonal conflict and reacting to it: the case for agreeableness. *J. Personal. Soc. Psychol.* 70 820–835. 10.1037/0022-3514.70.4.820 8636901

[B31] Greason P. B. Cashwell C. S. (2011). Mindfulness and counseling self-efficacy: the mediating role of attention and empathy. *Couns. Educ. Supervis.* 49 2–19. 10.1002/j.1556-6978.2009.tb00083.x

[B32] Hamilton J. D. (1994). *Time Series Analysis. Princeton University Press*. Available online at: https://ideas.repec.org/a/eee/intfor/v11y1995i3p494-495.html (accessed October 22, 2018).

[B33] Hubbard J. Mannell R. C. (2001). Testing competing models of the leisure constraint negotiation process in a corporate employee recreation setting. *Leisure Sci.* 23 145–163. 10.1080/014904001316896846

[B34] Kaplan D. (2016). *Meditation**: A Secret Superpower for Negotiation. Forbes.* Avaliable online at: https://www.forbes.com/sites/gradsoflife/2019/01/24/can-a-new-model-of-apprenticeships-save-whats-left-of-the-california-dream/#4d9b5f3d34b3 (accessed February 12, 2019).

[B35] Kelly E. J. Kaminskienë N. (2016). Importance of emotional intelligence in negotiation and mediation. *Int. Comp. Jurispr.* 2 55–60. 10.1016/j.icj.2016.07.001

[B36] Keng S.-L. Smoski M. J. Robins C. J. Ekblad A. G. Brantley J. G. (2012). Mechanisms of change in mindfulness-based stress reduction: self-compassion and mindfulness as mediators of intervention outcomes. *J. Cogn. Psychother.* 26 270–280. 10.1891/0889-8391.26.3.270 11261958

[B37] Kim K. Cundiff N. Bong Choi S. (2014). The influence of emotional intelligence on negotiation outcomes and the mediating effect of rapport. a structural equation. *Negotiat. J.* 30 49–68. 10.1111/nejo.12045

[B38] Kim K. Cundiff N. L. Choi S. B. (2015). Emotional intelligence and negotiation outcomes: mediating effects of rapport, negotiation strategy, and judgment accuracy. *Group Decis. Negot.* 24 477–493. 10.1007/s10726-015-9435-9

[B39] Kopelman S. Avi-Yonah O. Varghese A. K. (2012). “The mindful negotiator: strategic emotion management and wellbeing,” in *The Oxford Handbook of Positive Organizational Scholarship*, eds Spreitzer G. Cameron K. (London: Oxford University Press), 591–600.

[B40] Kuttner R. (2008). Wisdom cultivated through dialogue. *Negotiati. J.* 24 101–112. 10.1111/j.1571-9979.2007.00169.x

[B41] Kyl-Heku L. M. Buss D. M. (1996). Tactics as units of analysis in personality psychology: an illustration using tactics of hierarchy negotiation. *Personal. Individ. Differ.* 21 497–517. 10.1016/0191-8869(96)00103-1

[B42] Leventhal K. S. Gillham J. DeMaria L. Andrew G. Peabody J. Leventhal S. (2015). Building psychosocial assets and wellbeing among adolescent girls: a randomized controlled trial. *J. Adoles.* 45 284–295. 10.1016/j.adolescence.2015.09.011 26547145

[B43] Luque P. J. Medina F. J. Dorado M. A. Munduate L. (2014). Handling conflict styles effectiveness. *Int. J. Soc. Psychol.* 13 217–224. 10.1174/021347498760350713 25131292

[B44] Ma Z. (2008). Personality and negotiation revisited: toward a cognitive model of dyadic negotiation. *Manag. Res. News* 31 774–790. 10.1108/01409170810908525

[B45] Malhotra D. (2004). *Six Ways to Build Trust in Negotiations.* Boston, MA: Harvard Business School.

[B46] Mastenbroek W. F. (1987). *Conflict Management and Organization Development.* New York, NY: Wiley.

[B47] Mastenbroek W. F. (1991). “Development and negotiating skills,” in *International Negotiation: Analysis, Approaches, Issues* eds Kremenyuk V. A. (San Francisco: Jossey-Bass).

[B48] McClelland D. (1985). *Human Motivation.* New York, NY: Scott Foresman.

[B49] Miller O. (2014). The negotiation style: a comparative study between the stated and in-practice negotiation style. *Proc. Soc. Behav. Sci.* 124 200–209. 10.1016/j.sbspro.2014.02.478

[B50] Monteiro A. P. Serrano G. Rodríguez D. (2012). Conflict management styles, personality factors and effectiveness in the negotiation. *Int. J. Soc. Psychol.* 27 97–109. 10.1174/021347412798844042 25131292

[B51] Movius H. (2008). The effectiveness of negotiation training. *Negotiat. J.* 24 509–531. 10.1111/j.1571-9979.2008.00201.x

[B52] Mueller J. S. Curhan J. R. (2006). Emotional intelligence and counterpart mood induction in a negotiation. *Int. J. Confl. Manag.* 17 110–112. 10.1108/10444060610736602

[B53] Munduate J. L. Martínez R. J. M. (2003). *Conflicto y Negociación*, 2nd Edn Madrid: Pirámide.

[B54] Munduate L. Ganaza J. Alcaide M. (1993). Estilos de gestión del conflicto interpersonal en las organizaciones. *Rev. de Psicol. Soc.* 8 47–68. 10.1080/02134748.1993.10821669

[B55] Munduate L. Ganaza J. Peiro J. M. Euwema M. (1999). Patterns of styles in conflict management and effectiveness. *Int. J. Confl. Manag.* 10 5–24. 10.1108/eb022816

[B56] Munduate L. Medina D. J. (2013). *Gestión del Conflicto, Negociación y Mediación.* Madrid: Psicología Pirámide.

[B57] Murray G. Rawlings D. Allen N. Trinder J. (2003). NeoFive-factor inventory scores: psychometric properties in a community sample. *Meas. Eval. Counsel. Dev.* 36 140–149. 10.1080/07481756.2003.11909738

[B58] Neff K. (2003). Self-compassion: an alternative conceptualization of a healthy attitude toward oneself. *Self Identity* 2 85–101. 10.1080/15298860309032

[B59] Panke D. (2012). Negotiation effectiveness: why some states are better than others in making their voices count in EU negotiations. *Comp. Eur. Polit.* 10 111–132. 10.1057/cep.2011.3

[B60] Pimentel M. (2013). *Resolución de Conflictos: “Técnicas de Mediación y Negociación.* Barcelona: Plataforma Editorial.

[B61] Preuss M. van der Wijst P. (2017). A phase-specific analysis of negotiation styles. *J. Bus. Industr. Market.* 32 505–518. 10.1108/JBIM-01-2016-0010

[B62] Pruitt D. G. (2013). *Negotiation Behavior.* Cambridge,MA: Academic Press.

[B63] Pulido-Martos M. Lopez-Zafra E. Augusto-Landa J. M. (2013). Perceived emotional intelligence and its relationship with perceptions of effectiveness in negotiation. *J. Appl. Soc. Psychol.* 43 408–417. 10.1111/j.1559-1816.2013.01010.x

[B64] Rahim M. A. (1983a). A measure of styles of handling interpersonal Conflict. *Acad. Manag. J.* 26 368–376. 10.5465/255985 10263067

[B65] Rahim M. A. (1983b). *Rahim Organizational Conflict Inventory -II: Forms a, b, and c.* Palo Alto, CA: Consulting Psychologist Press.

[B66] Rahim M. A. Bonoma T. V. (1979). Managing organizational conflict: a model for diagnosis and intervention. *Psychol. Rep.* 16 143–155.

[B67] Reb R. Narayanan J. (2014). The influence of mindful attention on value claiming in distributive negotiations: evidence from four laboratory experiments. *Mindfulness* 5 756–766. 10.1007/s12671-013-0232-8

[B68] Riskin L. (2002). The contemplative lawyer: on the potential contributions of mindfulness meditation to law students, lawyers and their clients. *Harvard Negotiat. Law Rev.* 7 1–67.

[B69] Riskin L. L. (2004). Mindfulness: foundational training for dispute resolution. *J. Legal Educ.* 54 79–90.

[B70] Rodríguez J. C. Rodríguez-Mateo H. Luján I. (2017). Validez predictiva del pensamiento constructivo en la eficacia negociadora. *Int. J. Dev. Educ. Psychol.* 6 245–258.

[B71] Ruedy N. E. Schweitzer M. (2010). In the moment: the effect of mindfulness on ethical decision making. *J. Bus. Ethics* 95 73–87. 10.1007/s10551-011-0796-y

[B72] Salovey P. Mayer J. D. Goldman S. L. Turvey C. Palfai T. P. (1995). “Emotional attention, clarity, and repair: exploring emotional intelligence using the Trait Meta-Mood Scale,” in *Emotion, Disclosure, & Health*, ed. Pennebaker J. W. (Washington: American Psychological Association), 125–151.

[B73] Schlegel K. Mehu M. van Peer J. M. Scherer K. R. (2018). Sense and sensibility: the role of cognitive and emotional intelligence in negotiation. *J. Res. Personal.* 74 6–15. 10.1016/j.jrp.2017.12.003

[B74] Schneer J. A. Chanin M. N. (1987). Manifest needs as personality predispositions to conflict-handling behavior. *Hum. Relat.* 40 575–590. 10.1177/001872678704000903

[B75] Schneider A. K. (2002). Shattering negotiation myths: empirical evidence on the effectiveness of negotiation style. *Harv. Negot. L. Rev.* 7:143.

[B76] Schure M. B. Christopher J. Christopher S. (2008). Mind–body medicine and the art of self-care: teaching mindfulness to counseling students through yoga, meditation, and Qigong. *J. Counsel. Dev.* 86 47–56. 10.1002/j.1556-6678.2008.tb00625.x/abstract

[B77] Schutte N. S. Malouff J. M. (2011). Emotional intelligence mediates the relationship between mindfulness and subjective well-being. *Personal. Individ. Differ.* 50 1116–1119. 10.1016/j.paid.2011.01.037

[B78] Serrano G. Rodríguez D. (1993). *Negotiation in Organizations.* Salamanca: Eudema SA.

[B79] Sharma S. Bottom W. P. Elfenbein H. A. (2013). On the role of personality, cognitive ability, and emotional intelligence in predicting negotiation outcomes: a meta-analysis. *Organ. Psychol. Rev.* 3 293–336. 10.1177/2041386613505857

[B80] Sharma S. Elfenbein H. A. Foster J. Bottom W. P. (2018). Predicting negotiation performance from personality traits: a field study across multiple occupations. *Hum. Perform.* 31 145–164. 10.1080/08959285.2018.1481407

[B81] Shell G. R. (2001). Bargaining styles and negotiation: the thomas-kilmann conflict mode instrument in negotiation training. *Negotiat. J.* 17 155–174. 10.1023/A:1013280109471

[B82] Sudarsky J. Cleves J. (1976). Diseño de un instrumento para medir el perfil motivacional. *Revi. Latinoam. Psicol.* 8 425–477.

[B83] Taylor K. A. Mesmer-Magnus J. Burns T. M. (2008). Teaching the art of negotiation: improving students’ negotiating confidence and perceptions of effectiveness. *J. Educ. Bus.* 8 135–140. 10.3200/JOEB.83.3.135-140

[B84] Ten Velden F. S. Beersma B. De Dreu C. K. (2010). It takes one to tango: the effects of dyads’ epistemic motivation composition in negotiation. *Personal. Soc. Psychol. Bull.* 36 1454–1466. 10.1177/0146167210383698 20841436

[B85] Thomas D. C. (2006). Domain and development of cultural intelligence: the importance of mindfulness. *Group Organ. Manag.* 31 78–99. 10.1177/1059601105275266

[B86] Thompson L. L. Wang J. Gunia B. C. (2010). Negotiation. *Annu. Rev. Psychol.* 61 491–515. 10.1146/annurev.psych.093008.100458 19575611

[B87] Tokar D. Fischer A. Snell A. Harik-Williams N. (1999). Efficient assessment of the five-factor model of personality: structural validity analyses of the neo five-factor inventory (Form S). *Meas. Eval. Counsel. Dev.* 32 14–30. 10.1080/07481756.1999.12068967

[B88] Van der Schalk J. Beersma B. Van Kleef G. A. De Dreu C. K. (2010). The more (complex), the better? The influence of epistemic motivation on integrative bargaining in complex negotiation. *Eur. J. Soc. Psychol.* 40 355–365. 10.1002/ejsp.633

[B89] Velden F. S. T. Beersma B. De Dreu C. K. (2007). Majority and minority influence in group negotiation: the moderating effects of social motivation and decision rules. *J. Appl. Psychol.* 92 259–268. 10.1037/0021-9010.92.1.259 17227167

[B90] White D. D. (2008). A structural model of leisure constraints negotiation in outdoor recreation. *Leisure Sci.* 30 342–359. 10.1080/01490400802165131

[B91] Yiu T. W. Lee H. K. (2011). how do personality traits affect construction dispute negotiation? Study of big five personality model. *J. Constr. Eng. Manag.* 137 169–178. 10.1061/(ASCE)CO.1943-7862.0000271 29515898

